# Impact of *FLT3-ITD* location on cytarabine sensitivity in AML: a network-based approach

**DOI:** 10.1038/s41375-023-01881-5

**Published:** 2023-03-25

**Authors:** Giusj Monia Pugliese, Veronica Venafra, Valeria Bica, Giorgia Massacci, Sara Latini, Simone Graziosi, Thomas Fischer, Dimitrios Mougiakakos, Martin Boettcher, Livia Perfetto, Francesca Sacco

**Affiliations:** 1 Department of Biology, University of Rome Tor Vergata, Via della Ricerca Scientifica 1, 00133 Rome, Italy; 2 Institute of Molecular and Clinical Immunology, University of Magdeburg, Magdeburg, Germany; 3 Health Campus for Inflammation, Immunity and Infection (GCI3), University of Magdeburg, Magdeburg, Germany; 4 Department of Hematology and Oncology, University of Magdeburg, Magdeburg, Germany; 5 Department of Biology, Fondazione Human Technopole, Via Rita Levi-Montalcini 1, 20157 Milan, Italy; 6 Telethon Institute of Genetics and Medicine (TIGEM), Via Campi Flegrei 34, Pozzuoli, 80078 Italy

**Keywords:** Cell signalling, Acute myeloid leukaemia, Acute myeloid leukaemia

## To the Editor

25–30% of younger adults with newly diagnosed acute myeloid leukemia (AML) carry internal tandem duplications (ITDs) in the FLT3 gene [1]. ITD mutations regularly occur in exons 14 and 15 of FLT3, affecting JMD and TKD1 regions and causing conformational changes leading to the constitutive activation of the receptor tyrosine kinase [2]. These mutations are associated with poor prognosis and current therapeutic options include induction therapy (cytarabine and daunorubicine) followed by consolidation (cytarabine monotherapy) in combination with midostaurin, which is a tyrosine kinase inhibitor targeting FLT3 [3].

Combination of chemotherapy with FLT3 inhibitors has been shown to improve progression free survival in patients carrying the ITD in the JMD domain of FLT3. On the contrary, chemotherapeutic resistance has been associated with insertion of the ITD in the TKD region, which, therefore, represents an unfavorable prognostic factor for relapse and overall survival (OS) [2, 4]. Accordingly, we and others have demonstrated that ITD-TKD cell lines and primary mouse bone marrow cells show reduced apoptosis in response to different FLT3 inhibitors (i.e., gilteritinib and quizartinib) when compared to their ITD-JMD counterpart [5–8]. Moreover, recently we have also identified the molecular mechanisms driving resistant of ITD-TKD cells towards FLT3 inhibitors, further underlining the importance of a genotype-specific treatment for FLT3-ITD patients [8]. In contrast, impact of the distinct locations of the ITD-mutations on chemotherapeutic drugs sensitivity still remains a matter of current debate.

To address this question, we aimed at (i) investigating whether the ITD insertion sites influence responsiveness towards cytarabine (Ara-C) treatment in AML cell lines and patient-derived primary AML blasts, at (ii) elucidating the molecular mechanisms controlling Ara-C sensitivity, and at (iii) identifying novel potential therapeutic strategies to revert resistance in ITD-TKD AML cells.

**Fig. 1:**
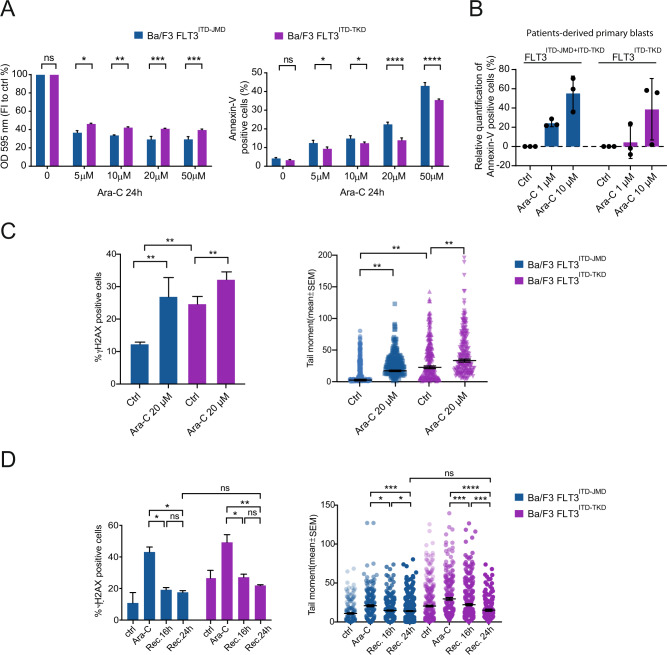
Impact of FLT3-ITD insertion site on apoptosis and DNA damage response. **A** Ba/F3 cells were incubated for 24 h with Ara-C. After incubation time cells were subjected to MTT and annexin-V assays to assess cells viability (left panel) and apoptosis (right panel), respectively. Data are presented as mean ± standard error (SE) from three independent experiments. *p < 0.05, **p < 0.01, ***p < 0.001, ****p < 0.0001; ANOVA test. **B** Relative quantification of treatment-induced apoptosis (100 * (dead cells after treatment – death cells in control) / viable cells in control) in patient-derived blasts carrying FLT3-ITD in both the JM and the TK1 domains (patients: #7, #10, #12) or in TK1 domain alone (patients: #2, #4, #9) upon treatment with Ara-C 1 µM and 10 µM for 24 h. **C** Ba/F3 cells were treated with 20 μM of Ara-C for 24 h. Then the amount of DNA damage was assessed by detection of γ-H2AX trough flow cytometry (left panel) and neutral comet assay (right panel). For each condition, 200 cells were scored using OpenComet plugin for ImageJ software (NIH) by calculating percentage of DNA in the comet tail. **p < 0.01; ANOVA test. **D** Recovery experiment was performed on Ba/F3 cells. Levels of DNA damage was assessed by monitoring the γ-H2AX level (left panel) and neutral comet assay (right panel) in control condition and upon treatment with Ara-C 20 μM for 24 h and after 16 h and 24 h upon treatment withdrawal. The bar plot shows the percentages of γ-H2AX-labeled cells (left panel) and DNA in the comet tail (right panel) obtained from three independent experiments. *p < 0.05, **p < 0.01, ***p < 0.001, ****p < 0.0001; ANOVA test.

To assess for differential drug sensitivity depending on ITD-location, we treated stably transfected hematopoietic Ba/F3 and 32D cells expressing the mutant FLT3 receptors carrying the ITD either in the JM or TK domains (FLT3^ITD-JMD^ and FLT3^ITD-TKD^) with Ara-C at clinically-relevant doses. In fact, FLT3^ITD-JMD^ cells were more susceptible towards in vitro Ara-C-mediated killing as compared to their FLT3^ITD-TKD^ counterpart (Fig. 1A). These effects were reproducible in two different cellular backgrounds (Ba/F3 and 32D cells) after 24 h of 20 µM Ara-C exposure (Fig. S1), confirming that the ITD-location determines sensitivity towards chemotherapy. Then, we investigated whether such distinct sensitivity could also be recapitulated in a setting closer resembling the clinical situation. To do so, we took advantage of a small cohort of FLT3-ITD AML patients, which we have previously classified in two main groups according to the ITD insertion site: 3 FLT3^ITD-TKD^ patients (carrying the ITD in the TKD domain) and 3 FLT3^ITD-JMD+ITD-TKD^ patients (carrying the ITD in the JMD and TKD domain) [8] (Fig. S2). Remarkably, genetic stratification based on the ITD localization reflected the drug-response phenotype: FLT3^ITD-JMD+ITD-TKD^ blasts showed higher sensitivity to Ara-C in cell death analysis, while induction of apoptosis was barely detected in FLT3^ITD-TKD^ blasts at 1 µM Ara-C (Fig. 1B). This result suggests that the pro-apoptotic effect of the ITD insertion within the JM domain is dominant over its ITD-TKD counterpart. This observation is consistent with recently published retrospective analyses showing that patients with insertions in JMD and/or in JMD and TKD1 had a significantly improved OS and a lower relapse rate as compared to patients with insertion sites in TKD1 [4].

Ara-C is an antineoplastic agent, which kills replicating cells by inhibiting DNA synthesis and generating DNA damage-double stranded breaks (DSBs) [9]. Given the role of FLT3-ITD in the regulation of DNA damage response genes [10, 11], we next investigated whether FLT3-ITD insertion sites may differently impact intrinsic and Ara-C-induced DNA damage. First, we assessed the levels of γ-H2AX, a widely used biomarker of DNA damage, in FLT3^ITD-JMD^ and FLT3^ITD-TKD^ cells under steady-state conditions. Interestingly, untreated FLT3^ITD-TKD^ cells show higher level of DNA damage as compared to FLT3^ITD-JMD^ cells, and as revealed by evaluating phosphorylated histone γ-H2AX (Fig. 1C, left panel) and neutral comet assays, that detect DSBs (Fig. 1C, right panel, Fig. S3). Increased intrinsic DNA damage has been linked to accumulation of reactive oxygen species (ROS). Indeed, when comparing intracellular ROS levels under steady state conditions, we found stronger ROS accumulation in FLT3^ITD-TKD^ cells (Fig. S4). Importantly, following Ara-C treatment, we observed that FLT3^ITD-TKD^ cells accumulated significantly less DNA damage than FLT3^ITD-JMD^ cells (fold change FLT3^ITD-JMD^ = 2.03, fold change FLT3^ITD-TKD^ = 0.94) (Fig. 1C).

Next, we asked whether differences in DNA damage seen in our two FLT3-ITD cell types correlated with a DNA repair efficacy. To examine the DNA repair efficacy, following 24 h Ara-C exposure, FLT3-ITD cells were cultured in drug-free medium for 0–24 h and DNA damage was analyzed at consecutive time points. Both γH2AX flow cytometry staining and comet assay showed similar results. Under steady-state conditions, FLT3^ITD-TKD^ cells showed higher levels of DNA damage than FLT3^ITD-JMD^ cells (Fig. 1D). Thereafter, DNA damage levels decreased as the extent of DNA repair was similar in both FLT3^ITD-JMD^ and FLT3^ITD-TKD^ cells (Fig. 1D). Taken together, our observations indicate that the FLT3-ITD insertion site impacts responsiveness to Ara-C treatment, without affecting DNA repair.

**Fig. 2:**
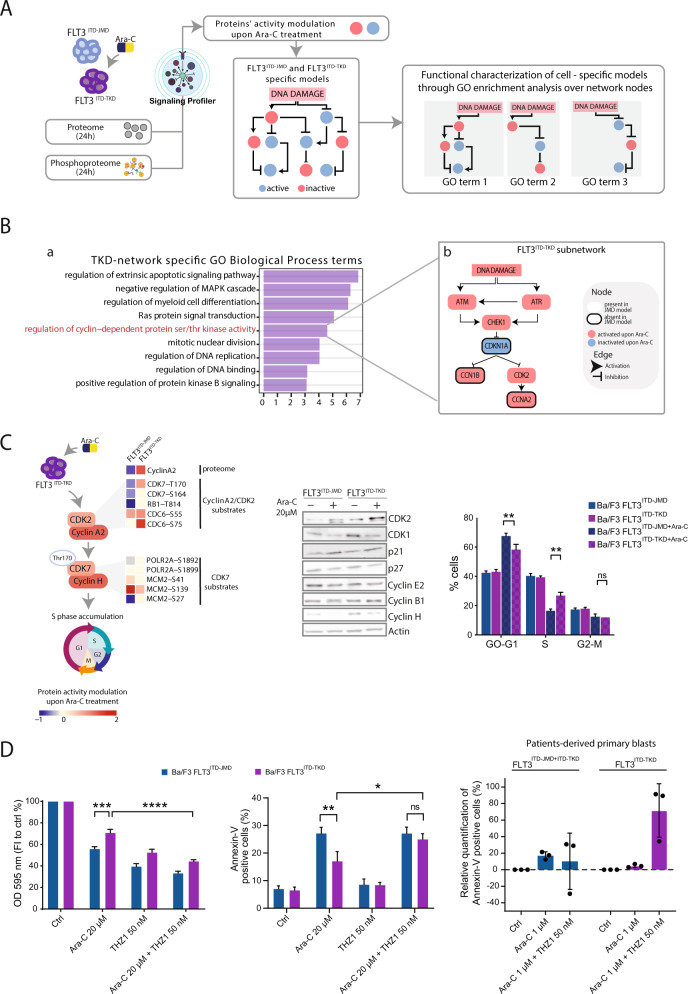
Combining MS-based phosphoproteomics and SignalingProfiler to identify the molecular mechanisms driving Ara-C response in FLT3-ITD cells. **A** Ba/F3 cells expressing FLT3^ITD-TKD^ (in purple) and FLT3^ITD-JMD^ (in blue) were treated with 20 μΜ Ara-C for 24 h and (phospho)proteome analysis was performed. We applied Signaling Profiler pipeline to obtain a list of proteins whose activity was altered upon Ara-C treatment (red activated, blue inactivated). Since Ara-C induces DNA damage, we connected modulated proteins to this phenotype to derive two FLT3 ITD Ara-C response specific models. We characterized models with pathway enrichment analysis and we derived functional circuits. **B** Barplot (a) showing the GO:BP terms enriched (Enrichment Score > 3) from FLT3^ITD-TKD^ model proteins list. FLT3^ITD-TKD^ specific mechanistic model (b) connecting DDR proteins to Ara-C modulated proteins belonging to ‘regulation of cyclin-dependent protein ser/thr kinase activity’ GO term. Black bordered nodes represent analytes absent in JMD model. **C** Left Panel Cartoon representing the potential molecular mechanism of drug resistance in FLT3^ITD-TKD^ cells. The heatmap shows the modulation of CyclinA2 in proteomic data and the modulation of phosphorylation levels of substrates (annotated in SIGNOR database) of CyclinA2/CDK2 complex and CDK7 kinase in phosphoproteomic data. FLT3^ITD-TKD^ cell line shows a higher expression of CyclinA2/CDK2 complex, leading to phosphorylation of CDK7 oh Thr170. Active CDK7, bound to cyclin H, promotes G1 – S cell cycle transition. Western blotting showing the levels of CDK2, CDK1, p21, p27 and of the indicated cyclins in FLT3^ITD-JMD^ and FLT3^ITD-TKD^ Ba/F3 cells upon treatment with Ara-C 20 µM for 24 h. Actin was used as loading control. Representative image of three independent experiments. Right panel, Cell cycle analysis was performed on Ba/F3 cells after 24 h of Ara-C 20 µM exposure. Flow cytometry-based cell cycle distribution was determined using DAPI staining. Each bar represents mean ± SE of the data obtained from three independent experiments. **p < 0.01; ANOVA test. **D** Ba/F3 cells (right and middle panels) and patients-derived primary blasts (left panel) were treated with Ara-C and/or THZ1 for 24 h. Cell viability and apoptosis were assessed by MTT and annexin-V assays. Left panel, relative quantification of treatment-induced apoptosis (100 * (dead cells after treatment – death cells in control) / viable cells in control) in patient-derived blasts carrying FLT3-ITD in both the JM and the TK1 domains (patients: #7, #10, #12) or in TK1 domain alone (patients: #2, #4, #9).

To elucidate the molecular mechanisms underlying the different sensitivity to Ara-C therapy in FLT3-ITD cells, we applied state-of-the-art, high-sensitive, mass spectrometry (MS)-based (phospho)proteomics followed by our recently developed modeling pipeline “SignalingProfiler” (Fig. 2A) (https://github.com/SaccoPerfettoLab/SignalingProfiler) [10]. This strategy enables us to obtain cell-specific models, that can be explored by performing gene ontology enrichment analysis [12].

As a first step in our approach, we have profiled the Ara-C-dependent modulation at the global proteome and phosphoproteome level in FLT3^ITD-TKD^ and FLT3^ITD-JMD^ cells by employing MS-based (phospho)proteomics. Using this strategy, about 4000 proteins (Table S1, Fig. S5A) and more than 13,000 phosphorylation events (class I sites, Table S2, Fig. S5B) were quantified. The biological triplicates were highly correlated with Pearson correlation coefficients ranging between 0.75 (for phospho measurements) (Fig. S5D) and 0.97 (for proteome measurements) (Fig. S5C).

Next, we applied a statistical *t*-test to narrow-down factors that are regulated by Ara-C treatment in the two FLT3-ITD mutant cell lines. Briefly, about 10% of the proteome and 15% phosphoproteome displayed a significant (FDR < 0.1) change upon Ara-C treatment (Tables S1, S2). Comparative analysis of significantly modulated proteins or phosphosites, revealed a common core (115 proteins and 89 phosphosites) of targets significantly altered by 24 h of Ara-C treatment, regardless of the ITD insertion site (Fig. S6A).

To elucidate the biological processes globally modulated by Ara-C treatment at the proteome layer, we performed an unsupervised hierarchical clustering that enabled us to separate the significantly modulated proteins in four main clusters (Fig. S6B; Table S2). We checked for biological processes altered in the four identified clusters by performing a gene ontology term enrichment analysis (FDR < 0.05) [13]. Interestingly, we found that Ara-C treatment positively regulates proteins significantly enriched in cell cycle and mitosis processes only in FLT3^ITD-TKD^ cells (Fig. S6B, bar graph).

Next, we used gene ontology term enrichment analysis to characterize the Ara-C-dependent modulation of the phosphoproteome in FLT3-ITD cells. From this analysis no differences between biological processes and pathways enriched in Ara-C treated FLT3^ITD-TKD^ and FLT3^ITD-JMD^ cells could be detected (Fig. S7A). On the contrary, our kinase substrate motif enrichment analysis revealed that cell cycle kinases, including CDKs and Aurora kinase, were significantly upregulated only in treated FLT3^ITD-TKD^ cells. Consistently with our proteomic data, core kinases involved in DNA damage response, including ATM, DNA-PK and CHK1, were similarly upregulated in Ara-C treated FLT3-ITD cells (Fig. S7B). To further corroborate our observations, we analyzed the levels of key proteins involved in DNA damage response. As shown in Fig. S8, Ara-C similarly trigger the DNA damage response pathway in FLT3-ITD cells, regardless of the ITD insertion site. This observation is consistent with the comparable ability of FLT3-ITD cells to repair damaged DNA (Fig. S7). In fact, these findings only provide a fragmented and vague picture of the processes involved in the cellular response to Ara-C, and point out the need for an unbiased, system-level approach able to produce more granular information regarding the molecular events involved. Thus, we exploited SignalingProfiler, a computational method that combines multi-omic data with literature-derived signaling networks of causal interactions to extract context-specific models of signal transduction [8]. Signaling Profiler first allowed us to infer the activity of 49 kinases, 4 phosphatases, 22 transcription factors and 156 other entities, in each cell line (Table S3). As shown in Fig. S7C, the correlation between the inferred activity of these signaling proteins in FLT3^ITD-JMD^ and FLT3^ITD-TKD^ is poor, suggesting that the different ITD insertion sites likely rewire signaling networks. Next, we derived FLT3^ITD-JMD^ and FLT3^ITD-TKD^ specific mechanistic models, consisting of 146 nodes and 183 edges (Figs. S9–S10), and we looked at the biological processes enriched by the proteins of the two models (Table S4) through gProfiler [12] (adjusted *p* < 0.05) (Table S4). Interestingly, this analysis resulted in the identification of cell cycle related terms upregulated in the FLT3^ITD-TKD^ model, confirming our previous findings [8] (Fig. 2B, panel a). A deep inspection of the generated models revealed that Ara-C treatment upregulates the CDK2-CCNA2 (Cyclin A2) only in FLT3^ITD-TKD^ cells (Fig. 2B, panel b). Thus, we speculated that CDK2-Cyclin A2 axis may play a role in Ara-C sensitivity by impacting cell cycle progression through the CDK7-Cyclin H activation (Fig. 2C, right panel).

First, we confirmed that in Ara-C treated FLT3^ITD-TKD^ cells expression of CDK2 and Cyclin H was significantly increased as compared to their FLT3^ITD-JMD^ counterparts (Fig. 2C, Fig. S11A). Consistently, our cell cycle analysis and EdU incorporation assay revealed that upon Ara-C treatment FLT3^ITD-TKD^ cells are significantly more accumulated in the S phase as compared to FLT3^ITD-JMD^ cells (Fig. 2C, left panel, Fig. S11B). Although Ara-C has been characterized as an S phase-specific chemotherapeutic drug, different cell types have been shown to be arrested in distinct phases of the cell cycle by Ara-C [14]. The differential cell cycle arrest has been shown to affect the severity of induced DNA damage and the pro-apoptotic efficacy. Therefore, we investigated whether pharmacological inhibition of CDK7 could increase the FLT3^ITD-TKD^ cells responsiveness to Ara-C treatment in both patients-derived FLT3-ITD primary blasts and Ba/F3 cells. As shown in Fig. 2D and S12A, our apoptotic and cell viability experiments show that Ara-C treatment in combination with THZ1, a highly selective CDK7 inhibitor, increases the sensitivity of FLT3^ITD-TKD^ cells. Additionally, our data suggest that while in FLT3^ITD-JMD^ cells Ara-C and THZ1 drugs seems to have a synergistic effect on apoptosis, in FLT3^ITD-TKD^ cells they have an additive effect (Fig. S12C).

Taken together, our findings confirm the clinical relevance of the FLT3-ITD location, which affects disease biology. In our study, ITD-location alters the intrinsic DNA damage level and sensitivity to cytarabine treatment in both AML cell lines and patient-derived primary AML blasts. We recently showed that different FLT3-ITD insertion sites alter sensitivity to TKIs treatment through the modulation of WEE1-CDK1 axis and cell cycle progression [8]. Applying the *SignalingProfiler* pipeline to our phosphoproteomic data revealed that Ara-C responsiveness is associated to the deregulation of the CDK2-CDK7 pathway and consequent accumulation of cells in the S phase. Pharmacological suppression of CDK7 synergistically acts with cytarabine, restoring the FLT3^ITD-TKD^ cells’ sensitivity. This observation is consistent with recent studies reporting that CDK7 inhibitor potentiates the anti-cancer effect of DNA damage agents or tyrosine kinase inhibitor as potent anti-cancer agents [15]. Finally, the ITD dependent differences in cell cycle progression may facilitate therapeutic strategies to sensitize FLT3^ITD-TKD^ cells to chemotherapy, however, these pathways need to be investigated more in depth in further pre-clinical studies.

## Supplementary information

Supplementary Material

Table S1

Table S2

Table S3

Table S4

## Data Availability

The mass spectrometry proteomics data have been deposited to the ProteomeXchange Consortium via the PRIDE partner repository with the dataset identifier PXD038638. Reviewer account details: Username: reviewer_pxd038638@ebi.ac.uk. Password: ckwYX9wz.
