# Supplementary material for: Impact of FLT3-ITD location on cytarabine sensitivity in AML: a network-based approach
Source: Leukemia. 2023 Mar 25;37(5):1151–5. doi: 10.1038/s41375-023-01881-5 (PMC10169656; doi:10.1038/s41375-023-01881-5)
Supplement: Supplementary file 1 — Supplementary Material [file 41375_2023_1881_MOESM1_ESM.pdf]

Supplementary material

Impact of *FLT3-ITD* location on cytarabine sensitivity in AML: a network-based approach.

Giusj Monia Pugliese[1*], Veronica Venafra[1*], Valeria Bica[1*], Giorgia Massacci[1], Sara Latini[1], Simone Graziosi[1], Thomas Fischer[2,3], Dimitrios Mougiakakos[4], Martin Boettcher[3,4], Livia Perfetto[1,4], Francesca Sacco[1,5#].

Supplementary Materials and Methods

Cell culture and chemicals

Murine 32D cells and Ba/F3 cells stably expressing JMD-ITD and TKD-ITD constructs were provided by courtesy of Prof. T. Fischer (1). The cells were cultured in RPMI 1640 medium (Thermo Scientific) supplemented with 10% heat-inactivated fetal bovine serum (EUROCLONE), 100 units/ml penicillin, 100 µg/ml streptomycin, 100X non-Essential Amino Acids (Thermo Scientific). The cells were grown at 37 °C in a humidified atmosphere containing 5% CO2. Peripheral blood (PB) samples from AML patients were obtained upon patient's informed consent and in accordance with the declaration of Helsinki (ethics committee approval number: 115/08). Mononuclear cells from the PB were obtained using Ficoll-Paque (GE Healthcare, Chicago, IL). Cryoconserved PBMCs from 6 FLT3-ITD patients were cultured at a density of 10^6 /mL in RPMI-1640 (Sigma-Aldrich, St. Louis, MO) supplemented with 10% FCS (c.c.pro, Germany), 2 mM L-glutamine (Sigma-Aldrich), and 40 U/mL Penicillin-Streptomycin (ThermoFisher Scientific) for 24h in absence or presence of increasing concentrations of Ara-C.

Ara-C (Selleck Chemicals, S1648) was used at 20 µM or as indicated in figures and dissolved in DMSO. THZ1 (Selleck Chemicals, S7549) was used at 50 nM and dissolved in DMSO.

Apoptosis analysis/assay

Apoptotic levels were analyze using Ebioscience™ Annexin V Apoptosis Detection Kit APC according to the kit instruction (Cat. 88-8007-74, Thermo Fisher Scientific). Briefly, Ba/F3 and 32D cell lines were treated with different doses of Ara-C for 24 hours. Then 10^6 cells were collected and washed in ice-cold PBS twice, and then resuspended in 1X binding buffer containing Annexin V for 15 minutes at RT°C in the dark. Apoptosis was measured by CytoFLEX using APC laser and analyzed by the CytExpert software.

Viability of the AML blasts was determined by flow cytometry using Annexin V – APC and 7AAD together with the Annexin V staining buffer according to the manufacturers' instruction (Biolegend, San Diego, CA). Prior to viability staining, samples were stained with fluorochrome-conjugated antibodies (see Supplemental table) after blocking with human IgG (Gamunex, Grifols, Barcelona, Spain). Samples were recorded on a Cytek NL-3000 spectral flow cytometer after unmixing. Data was analyzed using FlowJo V10 (Becton-Dickinson, Franklin Lakes, NJ). Specific cell death was calculated with the following formula to account for spontaneous, non-drug-related cell death: $\%\, specific\ cell\ death = 100 * (\frac{cell\ death - baseline\ cell\ death}{100 - baseline\ cell\ death})$.

Antigen	Fluorochrome	Clone	Manufacturer
**CD13**	PE	WM15	Biolegend
**CD33**	BV421	P67.6	Biolegend
**CD34**	BV510	581	Biolegend
**CD45**	BV711	HI30	Biolegend
**CD117**	PE-Cy7	104D2	Biolegend

Viability assays

Viability was assessed using the Cell Proliferation Kit I (MTT) (Roche). Cells were treated with a series of doses of Ara-C, or a combination as indicated, in a 96-well plate for 24 hours. MTT was added the least 4 hours of treatment and the cells were incubated at 37 °C. The formazan crystals were dissolved using the Solubilization Solution for O/N. The plates were read at 590 nm using a microplate reader (Bio-Rad).

(Phospho)proteomics sample preparation

Cell lysis was performed by adding SDC lysis buffer containing 4% (w/v) SDC, 100 mM Tris -HCl (pH 8.5). Samples were immediately boiled at 95° for 5 minutes and sonicated in bioruptor for 10 cycles at high intensity 30s on/30s off. Next, protein concentration was quantified by BCA assay. For the proteome preparation, we used the inStageTip (iST) method (2). Briefly, 50µg of protein for each sample was diluted in 2% SDC buffer and 1% t*rifluoroacetic acid* (*TFA*). SDBRPS tips were washed with i) 100 µl acetonitrile (ACN), ii) 100 µl of 30% methanol and 1% TFA and iii) 150 µl of 0.2 % TFA centrifuging tips at 1000 xg for 3 minutes. Samples were loaded onto equilibrated columns and spin at 1000 xg for 10 minutes. SDBRPS tips were washed with i) 100 µl of 1% TFA in ethyl acetate, ii) 100 µl of 1% TFA in isopropanol and iii) 0.2% TFA. Proteins were eluted with 80% ACN, 5% NH_4OH in MilliQ water. Samples were concentrated by SpeedVac at 45° for ~ 45 minutes. Finally, samples were dissolved in 10µl of a buffer containing 2% ACN and 0.1% TFA. Phosphoproteome preparation was performed by the EasyPhos workflow. 750 µg of protein was diluted in 750 µl of ACN and 250 µl of EP enrichment buffer containing: 36% TFA and 3mM KH_2PO_4. Samples were mixed at 2000 xg for 30s to clear precipitates and centrifuged at 20.000 xg for 15 minutes. To enrich phosphosites, TiO_2 beads were used. 12:1 (beads: protein) and resuspended in EP loading buffer containing 80% ACN and 6% (v/v) TFA. Next, beads were pelleted at 2000 xg for 1 minute and supernatants (non-phosphosites) were discarded. Beads were resuspended in 500µl of EP wash buffer consisting of 60% ACN, and 1% TFA. Beads were resuspended in 75 µl of EP transfer buffer (80%

ACN, 0.5% Acetic acid), transferred on top of C8 stage tips (double layer) and spin to dryness at 1000 xg for 5 minutes. Phosphopeptides were eluted with 30 µl of EP elution buffer containing 200 µl of NH_4OH and 800 µl of 40% ACN into PCR tubes. Immediately, samples were concentrated in SpeedVac at 45° C for 20 minutes. During SpeedVac, SDBRPS tips (triple layer) were equilibrated using i) 100 µl ACN, ii) 100 µl 30% methanol and 1% TFA and iii) 150 µl 0.2% TFA. After SpeedVac, SDBRPS loading buffer (1% TFA in isopropanol) was added to the samples. Next, phosphopetides were loaded onto equilibrated SDBRPS StageTips and washed with i) 100 µl 1% TFA in EtOAc, ii) 100 µl of 1% TFA in isopropanol and iii) 150 µl of 0.2% TFA. After wash phosphopetides were eluted into clean PCR tubes with a buffer containing 60% ACN and 5% NH_4OH. After SpeedVac at 45° C for 30 minutes, phosphopetides were resuspended in 10µl of a buffer containing 2% ACN and 0.1% TFA.

Mass spectrometry analyses

The peptides and the phosphopeptides were desalted on StageTips and separated on a reverse phase column (50 cm, packed in-house with 1.9-mm C18- Reprosil-AQ Pur reversed-phase beads) (Dr Maisch GmbH) over 120 min or 140 min (single-run proteome and phosphoproteome analysis respectively). After elution, peptides were electrosprayed and analyzed by tandem mass spectrometry on a Q Exactive Orbitrap (Thermo Fischer Scientific). Settings: 3E6 as AGC target, maximal injection time of 20 ms, and 120,000 resolution at 200 m/z. A data-dependent Top20 mode with sub sequent acquisition of higher-energy collisional dissociation (HCD) fragmentation MS/MS spectra of the top 20 most intense peaks. For MS/MS spectra, resolution: 15,000 at 200 m/z; 1E5 as AGC target; injection time: 20 ms; isolation window: 1.6Th.

MS Data processing

Raw mass spectrometry data were analyzed in the MaxQuant environment version 1.5.1.6, employing the Andromeda engine for database search. Proteome and phosphoproteome samples were analysed together by specifying two separate groups and setting group specific parameters for each sample type. MS/MS spectra were matched against the *Mus musculus* UniProtKB FASTA database (September 2014), with an FDR of < 1% at the level of proteins, peptides and modifications. Enzyme specificity was set to trypsin, allowing for cleavage N-terminal to proline and between aspartic acid and proline. The search included cysteine carbamidomethylation as a fixed modification. Variable modifications were set to N-terminal protein acetylation and oxidation of methionine as well as phosphorylation of serine, threonine tyrosine residue (STY) for the phosphoprotemic samples. MaxQuants Label free Quantification method and a minimum ration count of two was used for the

total proteome samples. For proteome and phosphoproteome analysis, where possible, the identity of peptides present but not sequenced in a given run was obtained by transferring identifications across liquid chromatography (LC)-MS runs. For phosphopeptides identification, an Andromeda minimum score and minimum delta score threshold of 40 and 17 were used, respectively. Peptides had to be fully tryptic in both proteome or phosphoproteme samples and up to two or four missed cleavages were allowed for protease digestion, respectively.

Proteome and Phosphoproteome Bioinformatics Data Analysis

Bioinformatic analysis was performed in the Perseus software environment (3). Statistical analysis of proteome and phosphoproteome were performed on logarithmized intensities for those values that were found to be quantified in any experimental condition. Phosphopeptides intensities were normalized by subtracting the median intensity of each sample. Student t-Test with a permutation-based FDR cutoff of 0.07 and S0 = 0.1 was performed to identify significantly modulated proteins and phosphopetides between two different conditions. Categorical annotation was added in Perseus in the form of GO biological process (GOBP), molecular function (GOMF), and cellular component (GOCC), KEGG pathways and kinase substrate motifs (extracted from HPRD). Concerning the kinase substrate motifs, we performed a 1D annotation enrichment analyses to identify statistically significant enriched kinase-substrates motifs (17). Multiple hypothesis testing was controlled by using a Benjamini-Hochberg FDR threshold of 0.05.

Signaling Profiler

The Signaling Profiler code and documentation is available as R package at https://github.com/SaccoPerfettoLab/SignalingProfiler/.

Step 1) Inference of protein activities modulation upon Ara-C treatment

We used three different methods in Signaling Profiler to infer the Ara-C induced activity modulation of key signaling proteins:

i) **VIPER score**: we used *run_footprint_based_analysis* function to infer the activity of kinases and phosphatases from phosphoproteomics data, setting analysis = 'ksea', reg_minsize = 1, exp_sign = FALSE (we consider the whole omic dataset) and correcting VIPER results through hypergeometric test (hypergeom_corr = TRUE);

ii) **PhosphoSCORE:** we used *phosphoscore_computation* function to infer the activity of phosphoproteins being target of (de)phosphorylation modification, setting organism = 'hybrid' to use human regulatory orthologs phosphosites in addition to mouse phosphosites;

iii) **Proteoscore:** we used *activity_from_proteomics* function to exploit the experimental fold-change in proteomic data as a proxy of activity modulation, setting organism = 'mouse'.

This process allowed us to predict the activity of 47 and 51 kinases, 4 and 5 phosphatases, 18 and 26 transcription factors and 158 and 154 other entities, in FLT3$^{ITD-JMD}$ and FLT3$^{ITD-TKD}$ cell line, respectively.

The complete list of inferred protein activities is provided in **Supplementary Table S3**.

Step 2) Cell – specific naïve causal network generation

We derived a naïve network from the Signaling Profiler built-in 'mouse' prior knowledge network (*choose_database_for_building*), connecting:

i) DNA Damage to inferred kinases and phosphatases through the *get_all_shortest_path_custom* function, setting as start_node_gn = 'DNA_DAMAGE', target_nodes_gn the list of inferred kinases and phosphatases and path_length = 'shortest'.

ii) kinases and phosphatases to their direct phosphorylated targets, using *get_all_shortest_path_custom*, setting path_length = 'one'.

Step 3) Cell specific causal network generation through CARNIVAL

We optimized the naïve network on inferred protein activities using CARNIVAL algorithm (4). In this step, we filter the naïve network retaining only causal paths coherent with the activity of start and end nodes. We exploited the *run_carnival_and_create_graph* Signaling Profiler function. We set DNA_DAMAGE as source node and we assigned the activity = 1, since Ara-C activates this phenotype. We set as target nodes all the inferred proteins present in the naïve network. We used as ILP solver *cplex* algorithm (4).

We obtained two cell – specific network linking DNA damage to key signaling Ara-C modulated proteins, namely the FLT3$^{ITD-JMD}$ model (139 nodes and 172 edges) and the FLT3$^{ITD-TKD}$ model (154 nodes and 195 edges).

The two networks are publicly available for browsing at:

- FLT3$^{ITD-JMD}$ model: https://www.ndexbio.org/viewer/networks/ec88c22e-3a55-11ed-ac45-0ac135e8bacf
- FLT3$^{ITD-TKD}$ model: https://www.ndexbio.org/viewer/networks/c08f9638-3a55-11ed-ac45-0ac135e8bacf

Step 4) Functional characterization of Ara-C specific models

The functional characterization of the proteins in FLT3 ITD Ara-C specific models was performed with gProfiler web tool (https://biit.cs.ut.ee/gprofiler/gost). We used as query the gene names of the proteins in each model, we set Organism to 'Mus musculus' and we selected as Data source only GO biological process.

Western blot analysis

Cells were seeded as indicated, then harvested, removed from culture medium and washed with ice-cold PBS. Cells were lysed in ice-cold lysis buffer (150 mM NaCl,50 mM Tris-HCl pH 7.5, 1% Nonidet P-40 (NP-40), 1 mM EGTA,5 mM MgCl2, 0.1% SDS) supplemented with 1 mM PMSF,1 mM orthovanadate, 1 mM NaF, protease inhibitor mixture1X, inhibitor phosphatase mixture II 1X, and inhibitor phosphatase mixture III 1X. The insoluble material was separated at 13,000 rpm for 30 min at 4 °C and total protein concentration was assayed on supernatants using Bradford reagent. Protein denaturation was performed with NuPAGE®LDS (Invitrogen). Then samples were heated at 95 °C for10 min. SDS-PAGE and transfer were performed on 4–15% Bio-Rad Mini PROTEAN®TGX™ and Trans-Blot®Turbo™ mini nitrocellulose membranes using a Trans-Blot®Turbo™ transfer System (Bio-Rad). Nonspecific binding sites were blocked using 5% non-fat dried milk in TBS-0.1% Tween-20 (TBS-T) for 1h at RT under shaking. Primary antibodies were diluted according to manufacturer's instruction and incubated overnight at 4 °C under shaking. HRP-conjugated secondary antibodies were diluted 1:3000 in 5% non-fat dried milk in TBS-T and incubated for 1h at RT under shaking. Immunolabeling was detected with Clarity™Western ECL Blotting Substrates (Bio-Rad) using the Las-3000 imaging system (Fujifilm). Densitometric quantification of bands was performed with Fiji (Image J, NIH). The primary antibodies used were: anti-pS1987ATM (1:1000, Millipore); anti-ATM (1:1000, Novus Biologicals); anti-BRCA1 (1:1000, Santa Cruz); anti-LigIII (1:1000, Proteintech); anti-pS824KAP1(1:1000, Bethyl); anti-KAP1 (1:1000, Bethyl); anti-RAD51 (1:1000, Abcam); anti-CDK1 (1:1000, Santa Cruz); anti p21 (1:1000, Santa Cruz); anti p27(1:1000, BD biosciences); anti-CDK2 (1:1000, Santa Cruz); the Cyclin Antibody Sampler Kit (Cell signaling); anti-RPA32 (1:1000, Cell signaling); anti-Actin (1:3000, Sigma Aldrich).

Flow Cytometry

Cells were treated with Ara-C for 24 hours and 10^6 cells were collected, washed twice with ice-cold PBS and fixed in 70% cold ethanol O/N. Next, cells were permeabilized by 1% saponin/1%BSA/PBS for 10 min. Next, the indicated antibodies were added, and the cells were incubated for 1 hour at RT°C. The primary antibodies used were: γH2AX (1:1000, Millipore). Alexa Fluor® 555 conjugated-goat anti mouse secondary antibody (Southern Biotech) was used at 1:200. Moreover, the blank tube single stained with secondary antibody was set. Then, where indicated, cells were resuspended in 1 μg/ml DAPI (Thermo Scientific, #62248) and 0.2 mg/ml RNase (Thermo Scientific, # 12091021) PBS solution before analysis.

In the end, the fluorescence intensity was detected using CytoFLEX S (Beckman Coulter). Quality control of the cytometer was assessed weekly using CytoFLEX Daily QC Fluorospheres (Beckman Coulter B53230). Data were collected by CytExpert (Beckman Coulter) software.

Neutral comet assay

DNA double strand break was evaluated by Neutral Comet assay (single-cell gel electrophoresis) in non-denaturing conditions. Briefly, cell pellets were resuspended in ice-cold PBS and kept on ice for all time. Cell suspensions were rapidly mixed with LMP agarose at 0.5% kept at 37 °C and an aliquot was pipetted onto agarose-covered surface of the slide. Agarose-embedded cells were lysed in lysis solution (30 mM EDTA, 0.1% sodium dodecyl sulfate (SDS)) and incubated at 4 °C, 40 min in the dark. After lysis, slides were washed in Tris Borate EDTA (TBE) 1X running buffer (Tris 90 mM; boric acid 90 mM; EDTA 4 mM). Electrophoresis was performed for 14 min in TBE 1X buffer at 15 V/cm. Slides were subsequently washed in distilled H2O and finally dehydrated O/N with ice-cold methanol. Nuclei were stained with Gel Red (1:1000, Biotium #41003) and visualized with a fluorescence microscope (LEICA), using a 20X objective, connected to a CCD camera for image acquisition. At least 200 comets per cell line were analyzed using Comet Assay Plug-in for ImageJ and data from tail moments processed using Prism software.

Cell cycle analysis

Cells were processed for flow cytometry as follows: for each point, 10^6 cells were collected, and after two washes in ice-cold PBS, fixed in 70% cold ethanol. Then, cells were washed in PBS/BSA 1% and then resuspended in1 μg/ml DAPI (Thermo Scientific, #62248) and 0.2 mg/ml RNase (Thermo Scientific, # 12091021) PBS solution before analysis. Data were detected using CytoFLEX S (Beckman Coulter) and analyzed by CytExpert (Beckman Coulter) software.

Statistical analysis

Data are represented as mean ± SE of at least three independent experimental samples (n=3). Comparisons between three or more groups were performed using ANOVA test. Statistical significance between two groups was estimated using Student's *t*-test. Statistical significance is defined as p value where *p< 0.05; **p<0.01; ***p< 0.001; ****p<0.0001. All statistical analyses were performed using Prism 7 (GraphPad).

Reactive oxygen species (ROS) detection

Reactive oxygen species (ROS) levels were analyze using CellROX™ Deep Red Flow Cytometry Assay Kit according to the kit instruction (Cat. C10491, Thermo Fisher Scientific). Briefly, CellROX (500nM) was added to cell cultures and incubated at 37°C for 60 minutes in the dark. $1x10^6$ cells were collected and washed in ice-cold PBS twice, then samples were immediately analyzed by flow cytometry. The data obtained were analyzed by CytExpert software.

Supplementary Figures

Figure S1

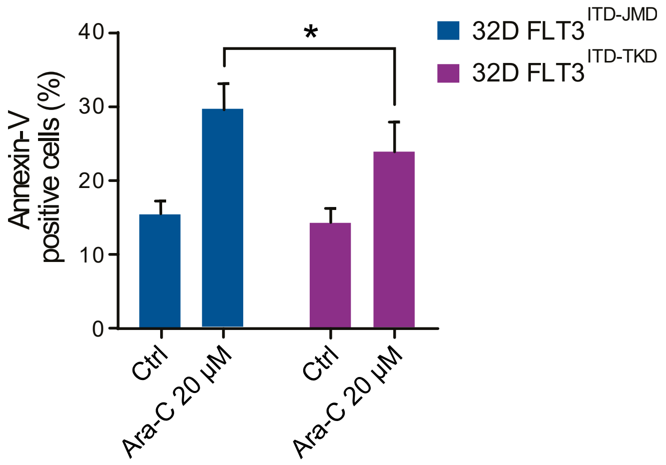

Figure S1. FLT3$^{ITD\text{-}TKD}$ mutation confers resistance to Ara-C exposure in 32D cell line. Annexin-V apoptosis assay of 32D cells exposed to 20 µM of Ara-C for 24h. Data are presented as percentage of apoptotic cells obtained from three independent experiments. Statistical analysis was performed by ANOVA test (*$p < 0.05$).

Figure S2

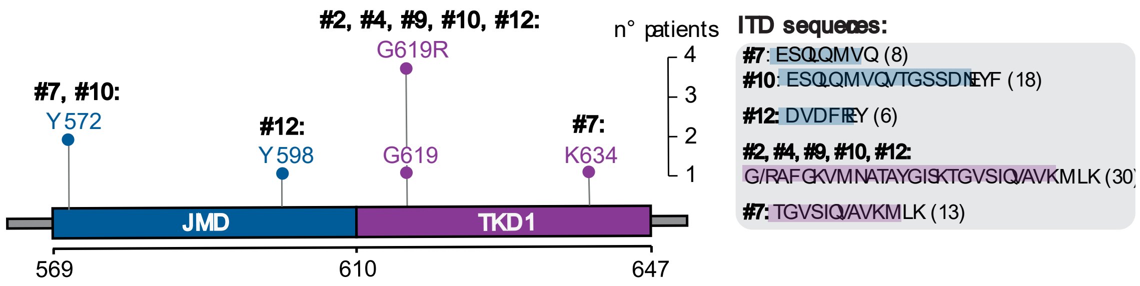

Figure S2. Lollipop plot representing the location, amino acid sequence and length of FLT3-ITD mutations in the 6 patients analyzed.

3 patients have ITD located in both TKD1 and JMD domain (#7, #10, #12) and 3 patients in TKD1 domain alone (#2, #4, #9). Each lollipop length represents the number of patients having an ITD in that position. All mutations were derived from Sanger sequencing from primary patient blasts.

Figure S3

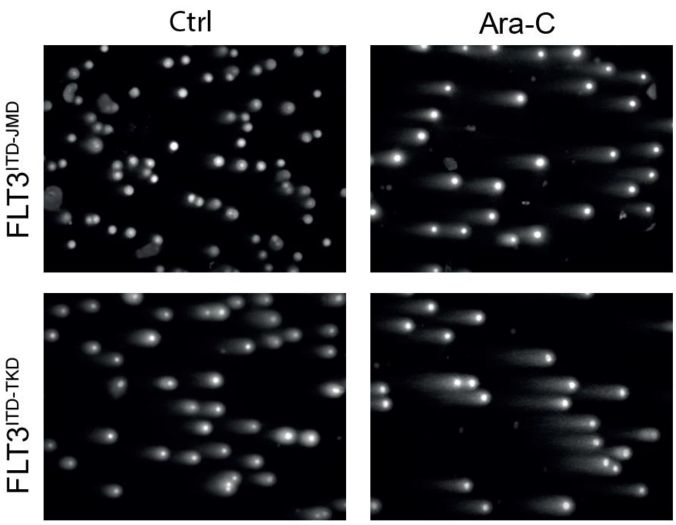

Figure S3. Ba/F3 FLT3$^{ITD-TKD}$ cells show higher levels of DNA damage after Ara-C exposure. Representative images of neutral comet assay on Ba/F3 FLT3$^{ITD-JMD}$ and FLT3$^{ITD-TKD}$ upon 20 μM Ara-C exposure for 24h.

Figure S4

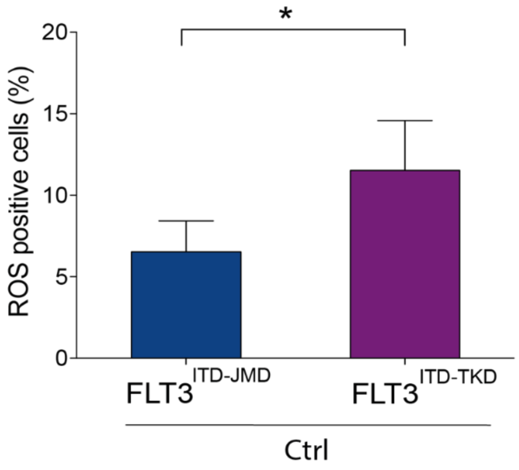

Figure S4. FLT3$^{ITD\text{-}TKD}$ cells show increased ROS level.

Bar plot showing the quantification of relative ROS level in Ba/F3 cells in untreated conditions. Data are represented as mean ± SE from three independent experiments performed in triplicate. *$p < 0.01$. Student *t*-test.

Figure S5

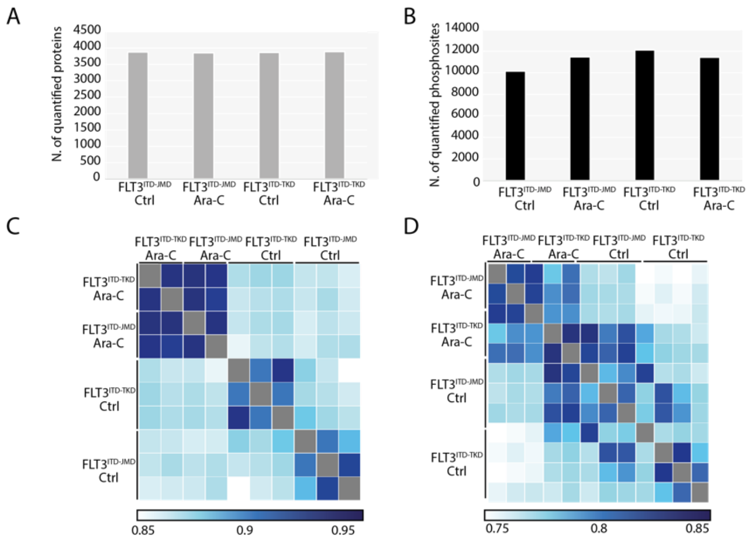

Figure S5. High coverage and reproducibility of proteome and phosphoproteome data.

(A-B) Average number of quantified proteins (A) and phosphosites (B) in Ara-C and control condition in FLT3$^{ITD-JMD}$ and FLT3$^{ITD-TKD}$ cells.

(C-D) Heatmap showing the Pearson correlation coefficients between different biological replicates in proteome (C) and phosphoproteome (D).

Figure S6

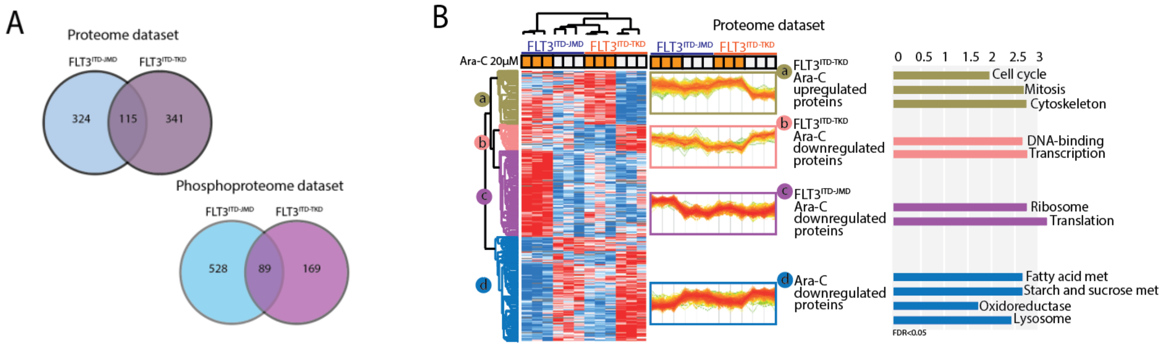

Figure S6.

(**A**) Venn diagram showing the overlap between significantly modulated proteins (upper panel) and phosphosites (lower panel) upon Ara-C treatment in FLT3$^{ITD-JMD}$ and FLT3$^{ITD-TKD}$. (**B**) Unsupervised hierarchical clustering representing the significantly modulated proteins (Log2 LFQ intensity is Z-scored). Pathways and GO-Biological processes significantly enriched in representative clusters are shown (right panel).

Figure S7

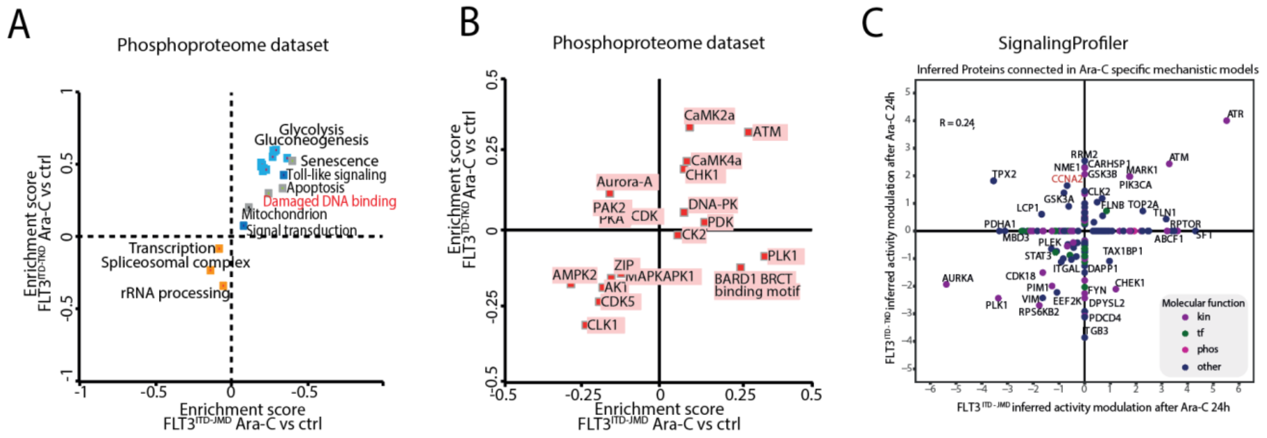

Figure S7.

(**A-B**) Scatterplot of pathways and GO-Biological processes (**A**) and kinase substrate motifs (**B**) significantly (FDR<0.05) enriched in FLT3$^{ITD-JMD}$ and FLT3$^{ITD-TKD}$ upon Ara-C treatment.

(**C**) Scatterplots showing the comparison between network protein activity predicted from FLT3$^{ITD-JMD}$ (x-axis) and FLT3$^{ITD-TKD}$ (y-axis) datasets for kinases (purple), phosphatases (violet), transcription factors (green) and other phosphoproteins (blue).

Figure S8

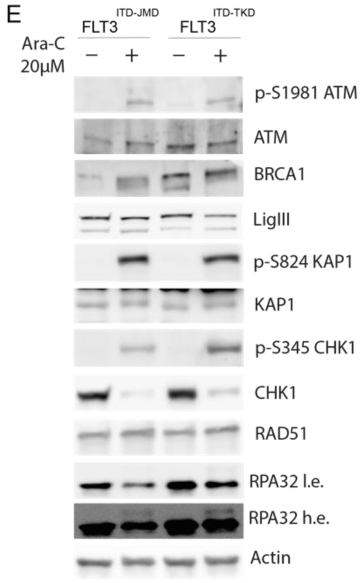

Figure S8. DDR pathway is not differently modulated in Ara-C treated FLT3-ITD cells. Whole cell lysates of Ba/F3 cells exposed to Ara-C 20 μM for 24h were subjected to Western blotting to measure DDR protein levels for the indicated antibodies. Actin was used as loading control. Representative images of three independent experiments are reported. l.e.= short exposure, h.e.= long exposure.

Figure S9

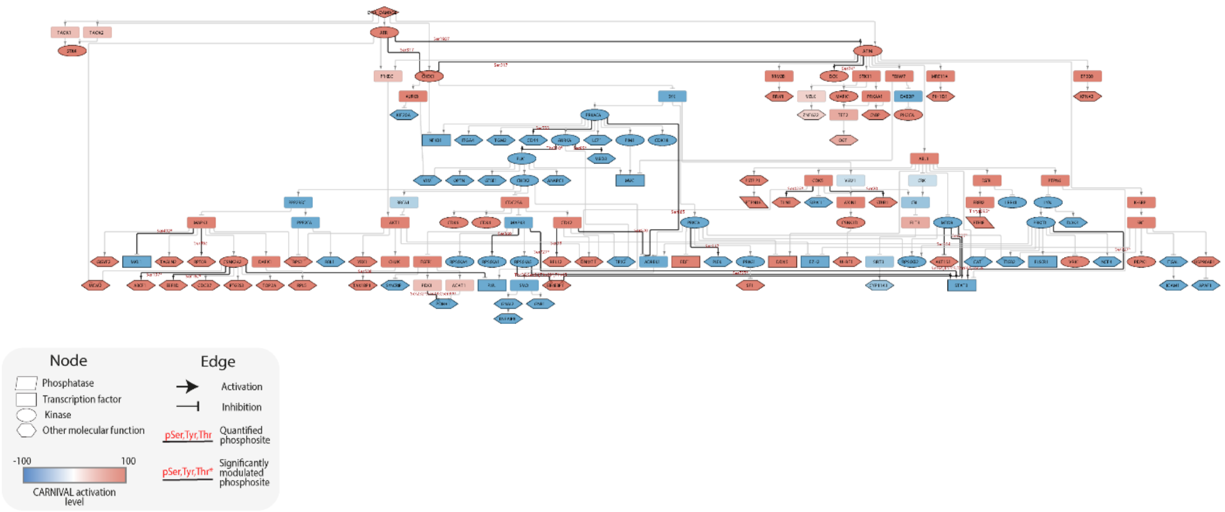

Figure S9. FLT3$^{ITD\text{-}JMD}$ specific causal network.

Causal network representing the Ara-C induced signal rewiring in FLT3$^{ITD\text{-}JMD}$ cell line. Color of nodes represents activated (red) or inhibited (blue) proteins after the treatment. Shape of nodes reflects molecular function: parallelograms are phosphatases, rectangles are transcription factors, circles are kinases and hexagons are other phosphorylated proteins. Target arrow shape represents activatory (arrow) or inhibitory (T shape) interactions. Black edges represent (de)phosphorylations occurring at phosphosites measured in the experimental data. Additional details are further described in the inset.

Figure S10

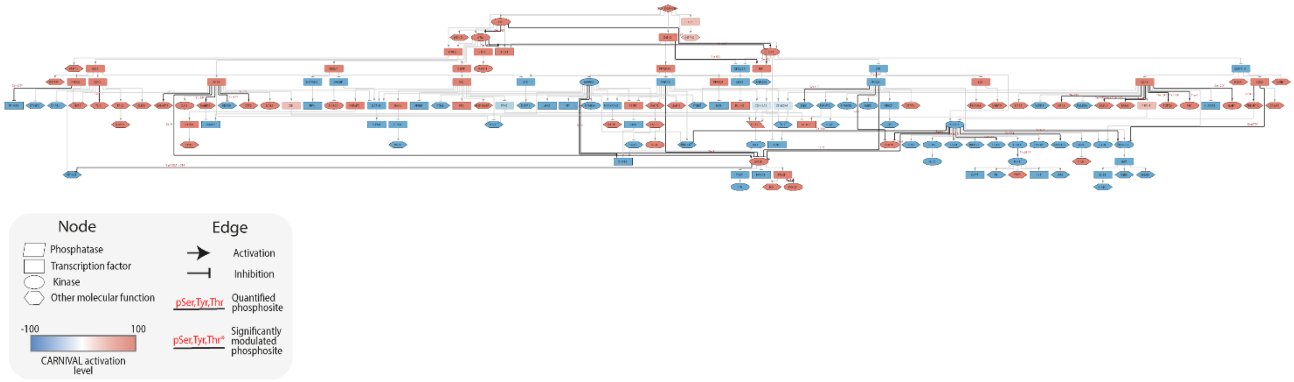

Figure S10. FLT3$^{ITD\text{-}TKD}$ specific causal network.

Causal network representing the Ara-C induced signal rewiring in FLT3$^{ITD\text{-}TKD}$ cell line. Color of nodes represents activated (red) or inhibited (blue) proteins after the treatment. Shape of nodes reflects molecular function: parallelograms are phosphatases, rectangles are transcription factors, circles are kinases and hexagons are other phosphorylated proteins. Target arrow shape represents activatory (arrow) or inhibitory (T shape) interactions. Black edges represent (de)phosphorylations occurring at phosphosites measured in the experimental data. Additional details are further described in the inset.

Figure S11

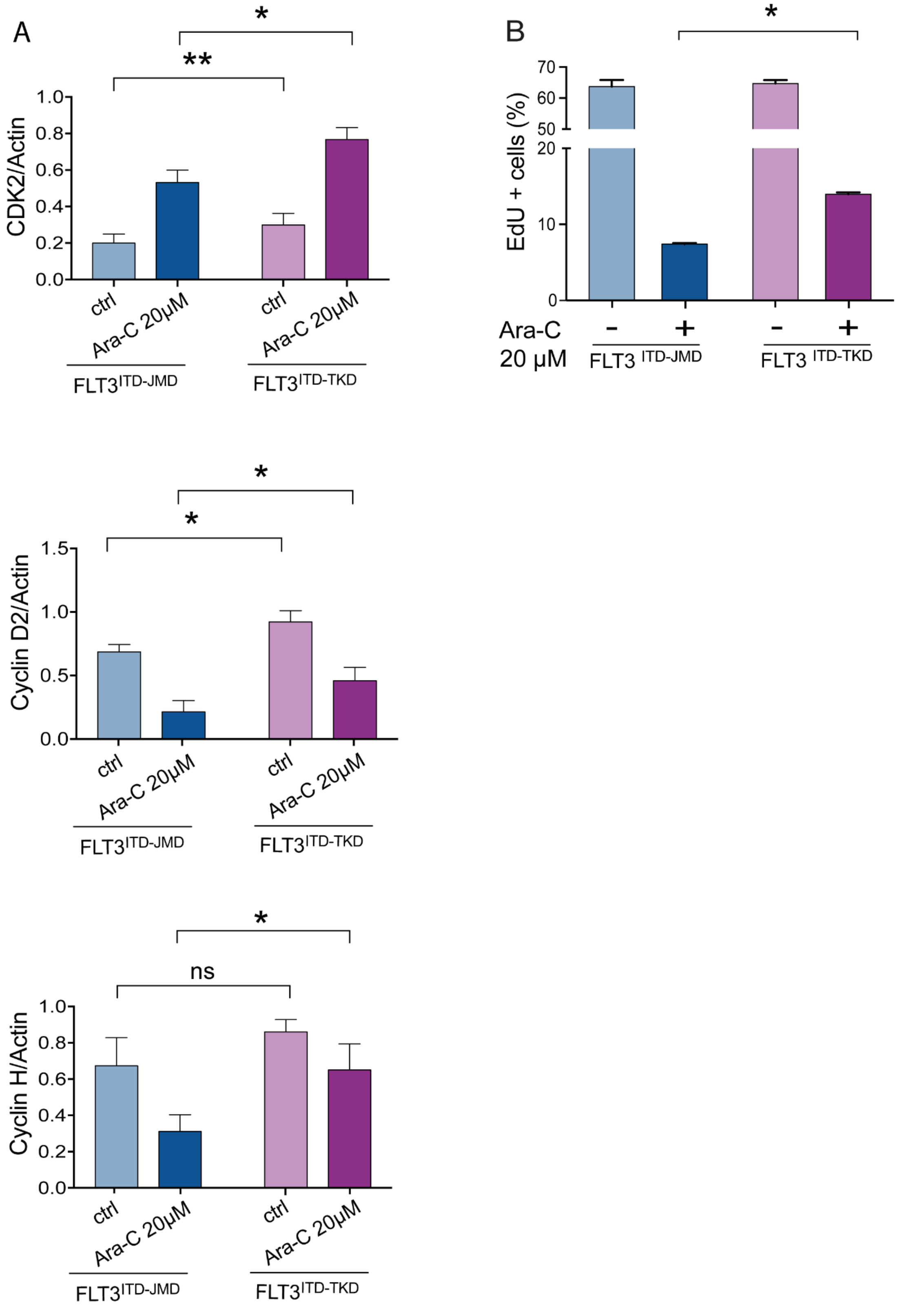

A
B
*
**
CDK2/Actin
1.0
0.8
0.6
0.4
0.2
0.0
ctrl
Ara-C 20μM
FLT3ITD-JMD
FLT3ITD-TKD
EdU + cells (%)
70
60
50
20
10
0
Ara-C
20 μM
-
+
FLT3 ITD-JMD
FLT3 ITD-TKD
Cyclin D2/Actin
1.5
1.0
0.5
0.0
Cyclin H/Actin
ns

Figure S11.

(A) Bar plot showing the protein expression level of the indicated protein normalized on Actin in $FLT3^{ITD\text{-}JMD}$ and $FLT3^{ITD\text{-}TKD}$ cells after 24h exposure to Ara-C 20 µM. *p < 0.05, **p < 0.01; ANOVA test.

(B) Replicating Ba/F3 cells were labelled with EdU for 30 min and then analyzed through flow cytometry. The graph shows the percentage of positive cells (S-phase) upon treatment with Ara-C 20 µM for 24h. Data are shown as mean ± SE from three independent experiments. *p < 0.05; ANOVA test.

Figure S12

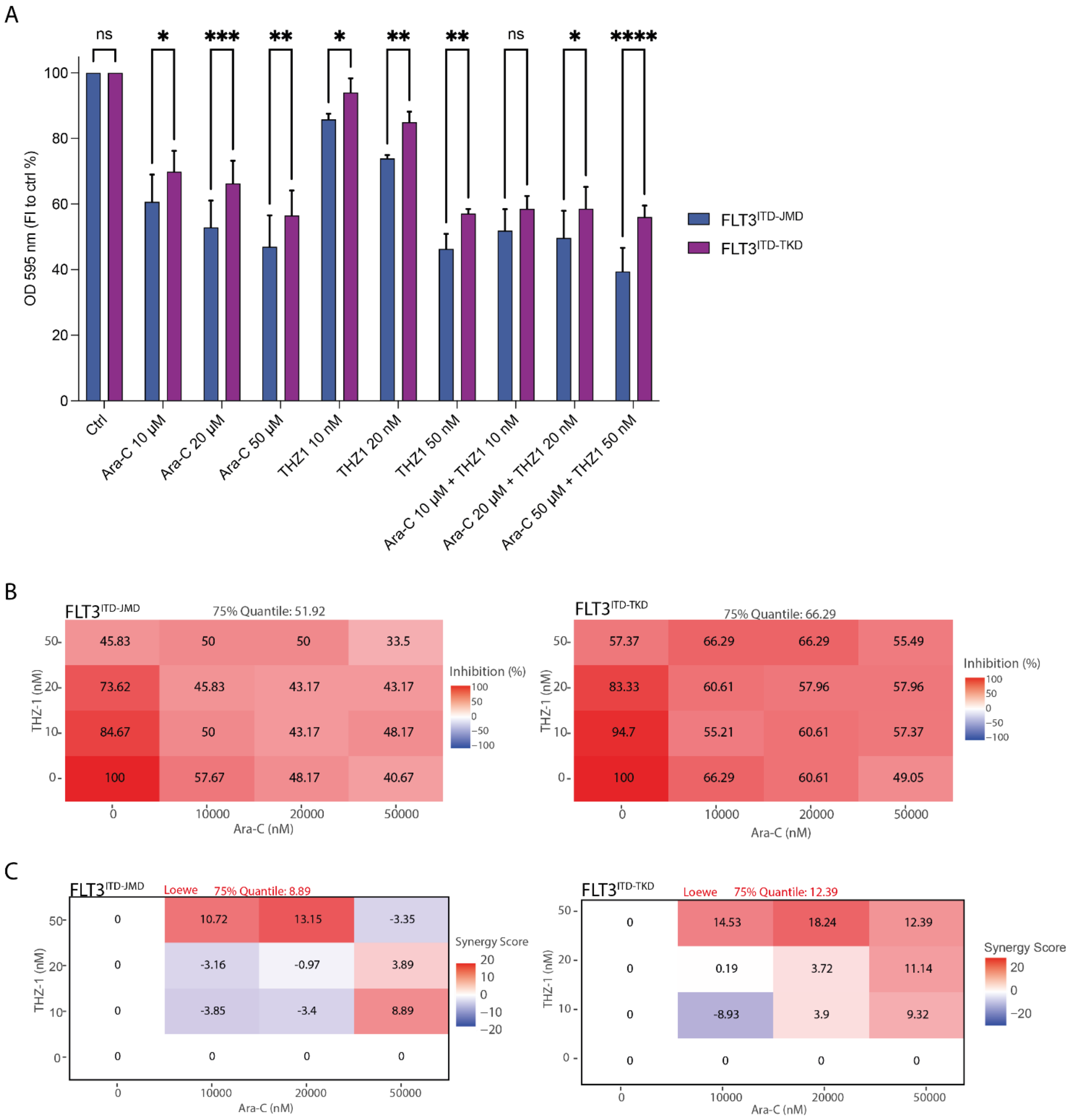

Figure S12. Synergy score.

A) Ba/F3 cells were treated with increasing doses of Ara-C and/or THZ1 for 24 hours. Cell viability was assessed by MTT assay. Data are presented as fold change on control condition of optical density measurements (OD = 595nm) obtained from three biological replicates. ***p < 0.001, ****p < 0.0001; ANOVA test. **B**) Heatmaps representing the dose response to Ara-C and THZ1 treatment in FLT3-ITD cells, generated by using the SynergyFinder tool. **C**) Heatmaps representing the synergy score of Ara-C and THZ1 drugs in FLT3-ITD cells, generated by using the SynergyFinder tool.

Supplementary Tables

Table S1. Quantified proteins in proteomic analysis (mass spectrometry).

Table S2. Quantified phosphopeptides in phosphoproteomic analysis (mass spectrometry).

Table S3. The complete list of protein activities inferred in the first step of "Signaling Profiler" pipeline.

Table S4. GO Biological Process terms enriched from list of proteins in FLT3 ITD-TKD specific model obtained with gProfiler.

References

1. Arreba-Tutusaus P, Mack TS, Bullinger L, Schnöder TM, Polanetzki A, Weinert S, et al. Impact of FLT3-ITD location on sensitivity to TKI-therapy in vitro and in vivo. Leukemia. 2016;30(5):1220–5.
2. Kulak NA, Pichler G, Paron I, Nagaraj N, Mann M. Minimal, encapsulated proteomic-sample processing applied to copy-number estimation in eukaryotic cells. Nat Methods. 2014;11(3):319–24.
3. Tyanova S, Temu T, Sinitcyn P, Carlson A, Hein MY, Geiger T, et al. The Perseus computational platform for comprehensive analysis of (prote)omics data. Nat Methods. 2016;13(9):731–40.
4. Liu A, Trairatphisan P, Gjerga E, Didangelos A, Barratt J, Saez-Rodriguez J. From expression footprints to causal pathways: contextualizing large signaling networks with CARNIVAL. npj Syst Biol Appl. 2019;5(1):1–10.
